# Increased sensitivity of *Aggregatibacter actinomycetemcomitans* to human serum is mediated by induction of a bacteriophage

**DOI:** 10.1111/omi.12378

**Published:** 2022-07-25

**Authors:** Gaoyan G. Tang‐Siegel, Casey Chen, Keith P. Mintz

**Affiliations:** ^1^ Department of Molecular Physiology and Biophysics, College of Medicine University of Vermont Burlington Vermont USA; ^2^ Ostrow School of Dentistry University of Southern California Los Angeles California USA; ^3^ Department of Microbiology and Molecular Genetics, College of Medicine University of Vermont Burlington Vermont USA

**Keywords:** bacteriophages, gram‐negative periodontal pathobionts, human serum, lytic cycle, Myoviridae, pseudolysogeny

## Abstract

*Aggregatibacter actinomycetemcomitans*, a Gram‐negative oral pathobiont causing aggressive periodontitis and systemic infections, demonstrates serum resistance. We have identified a dsDNA‐tailed bacteriophage, S1249, which was found to convert from this microorganism inducible by human serum into a lytic state to kill the bacterium. This phage demonstrated active transcripts when exposed to human serum: 20% of genes were upregulated more than 10‐fold, and 45% of them were upregulated 5–10‐fold when the bacterium was grown in the presence of human serum compared to without the presence of human serum. Transcriptional activation when grown in equine serum was less pronounced. This phage demonstrated a tail with inner rigid tubes and an outer contractile sheath, features of *Myoviridae* spp. Further characterization revealed that the lysogenized integration of the phage in the chromosome of *A. actinomycetemcomitans* occurred between the genes encoding cold‐shock DNA‐binding domain‐containing protein (*csp*) and glutamyl‐tRNA synthetase (*gltX*). Both phage DNA integrated lysogeny and nonintegrated pseudolysogeny were identified in the infected bacterium. A newly generated, lysogenized strain using this phage displayed similar attributes, including 63% growth inhibition compared to its isogenic phage‐free strain when in the presence of human serum. Our data suggest that bacteriophage S1249 can be induced in the presence of human serum and enters the lytic cycle, which reduces the viability of infected bacteria in vivo.

**Nomenclature**
CFUs: colony‐forming units
ORFs: open reading frames
TEM: transmission electron microscopy
PBS: phosphate buffered saline

## 1 INTRODUCTION

Gram‐negative *Aggregatibacter actinomycetemcomitans* is a causative agent of periodontitis and is mostly notable for its association with aggressive forms of the disease, which cause severe tissue loss in a relatively short period of time in young individuals (Casarin et al., 2010; Fine et al., 2018, 2020). This microorganism also causes infectious endocarditis (Anton‐Vazquez et al., 2022; Chambers et al., 2013), autoimmune diseases including rheumatoid arthritis (Konig et al., 2016), and neurodegenerative disorders driven by stimulation and release of proinflammatory cytokines (Hajishengallis & Chavakis, 2021).

*Aggregatibacter actinomycetemcomitans* has developed mechanisms to survive in serum‐rich environments in vivo, including resistance to complement‐mediated cell lysis and phagocytosis regardless of serotypes and leukotoxin production (Asakawa et al., 2003; Permpanich et al., 2006; Yamaguchi et al., 1995). These features allow it to survive in hostile environments of periodontal pockets and blood circulation, where serum is the predominant nutrient source for bacterial metabolism (Asakawa et al., 2003; Sundqvist & Johansson, 1982). However, our work indicates that selective clinical strains of *A. actinomycetemcomitans* display serum sensitivity (Tang‐Siegel et al., 2016), including D11S‐1, which demonstrated an 85% reduction in the number of recovered colony‐forming units (CFUs) in the presence of human serum compared to other strains (Tang‐Siegel et al., 2016). Genomic DNA sequence analyses of D11S‐1 revealed the presence of a bacteriophage, noted as S1249 (Chen et al., 2009; Tang‐Siegel et al., 2016, 2021). In the presence of serum, this phage is induced to undergo a transition from a lysogenized prophage into the lytic cycle, resulting in bacterial lysis (Tang‐Siegel et al., 2021).

In this study, we isolated, characterized, and developed a methodology to study phage infection physiology in this oral pathobiont. The morphology of the isolated phage was determined by negatively stained imaging of the isolated bacteriophage using transmission electron microscopy (TEM), which was observed to be a contractile tailed phage. Interestingly, circular phage DNA was found either free in the bacterial cytosol or integrated into the bacterial chromosome. Infection with a naïve, serum‐resistant strain resulted in an ∼60% increase in serum sensitivity. Transcriptomics of the phage following exposure to sera revealed increased levels of transcription in all 66 phage open reading frames (ORFs). However, the increased amounts were not as pronounced as those in human serum when the same strain was grown in equivalent concentrations of equine serum. An increased amount of phage DNA was also observed in the spent culture medium following bacterial incubation in human serum. Together, this study suggests that the increased serum sensitivity of strain D11S‐1 and newly infected strains is related to the induction of bacteriophage S1249 by certain human serum components, which may be used for the development of an alternative therapeutic agent to control bacterial infection in vivo.

## 2 METHODS

### 2.1 Bacterial strains

All strains (Table 1) were stored at −80°C. *Aggregatibacter actinomycetemcomitans* was grown on TSBYE medium containing 3% trypticase soy broth, 0.6% yeast extract with/without 1.5% agar (Becton Dickinson and Company), and incubated statically in a 37°C incubator with 5% humidified carbon dioxide.

**TABLE 1:** Bacterium and phage strains

Strains	Genotype/remarks	Sources and references
*A. actinomycetemcomitans*		
D11S‐1	Serotype c, isolated from a 16 year African with GAP, lysogen of *Aggregatibacter* S1249	C. Chen et al. (2009), Tang‐Siegel et al. (2016)
D7S‐1	Serotype a, isolated from a 29 year African with GAP	C. Chen et al. (2010), Tang‐Siegel et al. (2016)
SCC1398	Serotype b, isolated from a 25‐year Caucasian with LAP	Tang‐Siegel et al. (2016)
IDH84	A serotype c strain isolated from human oral cavity, Parent strain of IDH84/S1249	Tang et al. (2007)
IDH84/S1249	*Aggregatibacter* S1249 lysogenized IDH84	This study
ATCC29523	*Aggregatibacter* S1249 is lytic in this strain without lysogenization	ATCC
*Myoviridae*		
*Aggregatibacter* S1249	A contractile tailed phage isolated from D11S‐1, 44 kb linear double strand DNA virus	This study

Abbreviations: ATCC, American Type Culture Collection; GAP, generalized aggressive periodontitis; LAP, localized aggressive periodontitis.

### 2.2 Phage isolation

*Aggregatibacter* phage S1249 was isolated from clinical strain D11S‐1 (C. Chen et al., 2009) and stored in phage buffer (0.7% Na_2_HPO_4_, 0.3% KH_2_PO_4_, 0.5% NaCl, 1 mM MgSO_4_, 0.1 mM CaCl_2_) at −80°C (Stevens et al., 1982). Briefly, 10 ml of overnight culture was grown as described above in modified thioglycolate broth (1.5%trypticpeptone, 0.5%yeast extract, 0.75% dextrose, 0.25% sodium chloride, 0.075% l‐cysteine, 0.05% sodium thioglycolate, and 0.4% sodium bicarbonate) (Stevens et al., 1982). The culture was diluted in 1:10, grown for two doubling times (6 h), and exposed to 0.5 μg/ml mitomycin C (M0503; Millipore Sigma, Burlington, MA, USA) for 30 min (F. Chen et al., 2006). Cells were then collected by centrifugation at 4°C and 8000 × *g* for 10 min, washed twice with thioglycolate broth, and resuspended in 100 ml fresh broth. After overnight recovery, the culture was first centrifuged at 4°C and 16,000 × *g* to pellet the bacterium, and the collected supernatant was filtered and ultracentrifuged at 4°C and 100,000 × *g* for 1 h to pellet the phage. The isolated phage was resuspended in phage buffer, stored at −80°C and confirmed to be bacterium‐free before use for the infection of other strains.

### 2.3 TEM analysis of phage S1249

Isolated phages were examined under TEM. Approximately 5 μl of the prepared phage sample was diluted in phosphate buffered saline (PBS; 10 mM sodium phosphate, 150 mM sodium chloride, pH 7.4), transferred to a 200‐mesh carbon‐coated nickel grid, washed with PBS, and negatively stained with 2% methylamine tungstate, Nano W (Nanoprobes, Yaphank, NY, USA). Grids were imaged using a JOEL electron microscope (Peabody, MA, USA).

### 2.4 DNA and RNA isolation

Bacterial strains were inoculated from TSBYE agar plates and transferred to liquid TSBYE medium in polystyrene culture tubes without serum and grown overnight, and adherent cells were subsequently exposed to fresh TSBYE media with or without the addition of either 50% pooled, male type AB heat‐inactivated human serum (H3667; Sigma‒Aldrich, St Louis, MO) or 50% heat‐inactivated equine serum (262‐500; Quad Five, Ryegate, MT, USA) as a control. Attached bacterial cells were removed from the sides of individual polystyrene culture tubes using cell scrapers every doubling time (3 h) over a 24‐h period. Total DNA was extracted from cells grown under each of three growth conditions (TSBYE, TSBYE with 50% equine serum, TSBYE with 50% human serum) collected at different time points using the QIAamp DNA kit (Qiagen, Germantown, MD, USA).

Total RNA was also extracted from bacteria after 6 h of growth using a bacterial RNA isolation kit based on the manufacturer's instructions (Ribo‐Pure, Life Technology, Grand Island, NY, USA). Briefly, 1.0 × 10^9^ cells were lysed using zirconia beads, and the lysate was mixed with chloroform. The RNA was extracted in the top aqueous phase, cleaned, eluted in 100 μl RNase‐free water, and treated with 8 U of DNase (Ribo‐Pure, Life Technology) at 37°C for 30 min, followed by DNase inactivation at room temperature for 2 min. The DNase inactivation reagent was pelleted by centrifugation, and the total RNA in the supernatants was collected (Tang‐Siegel et al., 2016).

### 2.5 Transcriptomic analysis

Total RNA was further purified to remove ribosomal RNA using the Ribo‐Zero Magnetic Kit (Illumina, San Diego, CA, USA) (Tang‐Siegel et al., 2016). Briefly, approximately 1.0 μg total RNA was mixed with rRNA removal solution and incubated at 68°C for 10 min at room temperature for 5 min, followed by mixing with prepared magnetic beads with the RNase inhibitor, incubating at room temperature for 5 min, and vortexing at 50°C for 5 min. The sample was placed in a magnetic stand, and the rRNA‐depleted supernatant was further precipitated with ethanol and eluted in RNase‐free water. The prepared rRNA‐depleted sample was fragmented using divalent cations at elevated temperature. Cleaved RNA fragments were copied into first strand cDNA using reverse transcriptase and random primers, followed by second strand cDNA synthesis using DNA polymerase I and RNase H. cDNA products were purified and enriched by PCR to create a final cDNA library according to the TruSeq stranded total RNA sample preparation kit (Illumina,) (Tang‐Siegel et al., 2016). After sequencing, the reads for each sample were mapped to the phage genomes using Geneious software (Biomatters LTD, Auckland, New Zealand). After mapping, the average coverage (number of sequences/nucleotide) was calculated for each predicted gene. Coverage was normalized by averaging across all genes for each sample and scaled up by multiplying by a factor of 1000 (Tang‐Siegel et al., 2016). All transcriptomic data were generated based on duplicate experiments.

### 2.6 Phage kinetics in *A. actinomycetemcomitans* grown in human serum‐containing medium

Strain D11S‐1 was inoculated from TSBYE agar to liquid TSBYE culture medium using polystyrene culture tubes and grown overnight. The adherent cells were exposed to fresh TSBYE media with 50% human serum. Bacterial culture spend media were collected every 3 h over a 24‐h period, centrifuged at 4°C, 8000 × *g*, and filtered with a 0.22 μm membrane, and 3 μl of the filtered spent medium was used for semiquantitive analysis of phage virion release by PCR with two sets of primers specific for the phage gene *bpp* (baseplate protein) and *tmp* (tail length tape measure protein) (Table 2). The phage amplicons from three independent experiments were quantified using ImageJ (https://imagej.nih.gov/ij/download.html). Statistical analyses were performed using ANOVA, and *p* < 0.05 was considered significant.

**TABLE 2:** Primers used in this study

Name	Sequences (5′−3′)	Genes/ORFs
*tmp*_F	AAGCAAAGGAAATGCCTGAA	Tail length tape measure protein (*tmp*) of phage, D11S_2220
*tmp*_R	AATCCAAGGGAAACCAAACC	*Tmp*
*bpp*_F	ATGACCAATCGCTTGCTACC	Baseplate protein (*bpp*) of phage, D11S_2215
*bpp*_R	AACTTACCGGCACATCAACC	*Bpp*
*in*t_F	ATGTCACGTCAAATCATCTCC	Integrase (*int*) of phage
*int*_R	TTAGTTATTGATAAGGTTTTTTCCTCT	*Int*, D11S_2273
*int*_F2	GCAGCAATTAGCCTCAAAGG	*int*
*attP*_F	GATCGAACCGACGACCTCT	Part of the 49‐bp *attP*/*attB* sequence
ORF1_R	TCGTGGCAATCGATCAGTTA	D11S_2208, the first ORF of phage
*csp_*F	GTGGGAGCGATAAAACCAAA	Cold‐shock DNA‐binding domain‐containing protein (*csp*) of *A.a*.
*gltX*_R	GAAGGTTCCGTGGTTTTTGA	Glutamyl‐tRNA synthetase (*gltX*) of *A.a*.

Abbreviations: A.a., *A. actinomycetemcomitans*; ORFs, open reading frames.

The corresponding bacterial culture spent media were also collected by low‐speed centrifugation 8000 × *g* to remove the pellets of cells, filtered with a 0.22 μm membrane, and ultracentrifuged at 100,000 × *g* to pellet the released virions, resuspended in phage buffer and prepared for TEM. Approximately 5 μl of sample was diluted in PBS at pH 7.4, transferred to a 200‐mesh carbon‐coated nickel grid, washed a couple of times with PBS, and negatively stained with 2% methylamine tungstate, Nano W (Nanoprobes). Grids were imaged using a JOEL electron microscope (Peabody).

### 2.7 Determination of phage DNA integration within the bacterial chromosome

Two primers (*csp*_F, *gltX*_R) specific for bacterial genes of cold‐shock DNA‐binding domain‐containing protein (*csp*) and glutamyl‐tRNA synthetase (*gltX*) and two primers amplifying the 1 kbp integrase gene (*int*_F and *int_*R) of the phage were designed (Table 2) based on the phage attachment sites (*attP*) identified from phage S1249 and the bacterial attachment site (*attB*) identified from *A. actinomycetemcomitans* (C. Chen et al., 2009; Resch et al., 2004). The chromosome DNA of D11S‐1 was extracted from overnight growth of one colony in 5 ml TSBYE using the QIAamp DNA kit (Qiagen) and prepared for PCR. Briefly, a total 5 ml culture of approximately 10^9^ cells was pelleted and resuspended in 180 μl of buffer ATL, mixed with proteinase K and incubated at 56°C overnight with occasional vortexing. The digested samples were added to 200 μl buffer AL, incubated at 70°C for 10 min, and subjected to the standard protocol using spin columns to isolate DNA. The phage DNA was isolated from the filtered spent medium of D11S‐1 after 6 h of growth in 50% human serum using the QIAamp DNA kit (Qiagen) following the protocol of DNA purification from blood or body fluid. Multiplex single colony PCR was also performed using four primers (*csp*_F, *gltX*_R, *int*_F, and *int_*R) at the same time to locate the phage DNA in the bacterium.

### 2.8 Phage specificity and genotypic and phenotypic analyses of newly infected strains

A total of seven strains representing serotypes a to f were chosen for phage infection. Strains were recovered from −80°C to TSBYE agar plates, and a single colony was transferred into 10 ml TSBYE broth, grown overnight, diluted in 1:10, and grown one doubling time before pelleting bacterial cells. A ratio of ∼100:1 (bacterium: phage) was used for cell infection. Cells were diluted in 50 μl TSBYE and mixed with 50 μl phage diluted in phage suspension medium (100 mM NaCl, 8 mM MgSO4.7H_2_O, 0.01% gelatin, 50 mM Tris‐HCl) (Sørensen et al., 2015) and incubated at room temperature for 30 min before spreading on TSBYE plates. A negative control was performed by using bacterial cells alone. Colony PCR was performed using multiple primers specific for the *bpp*, *tmp*, and *int* genes from the phage (Table 2). The phage‐positive colony was subcultured again to confirm the stability of phage infection.

The genotypes and growth phenotypes of newly infected strains versus their isogenic phage‐free strains were evaluated by growing the bacteria in TSBYE and TSBYE containing 75% pooled, male type AB, nonheated, human serum (H4522, Sigma‒Aldrich). Bacteria were collected after 6 and 23 h of growth, diluted to the proper concentration and plated on TSBYE agar medium. After 3 days of growth, CFUs were determined. Statistical analyses were performed based on triplicate experiments, paired samples were analyzed using Student's *t*‐test, and *p* < 0.05 was considered significant.

## 3 RESULTS

### 3.1 Phage morphology and sequence alignment analyses

**FIGURE 1:**
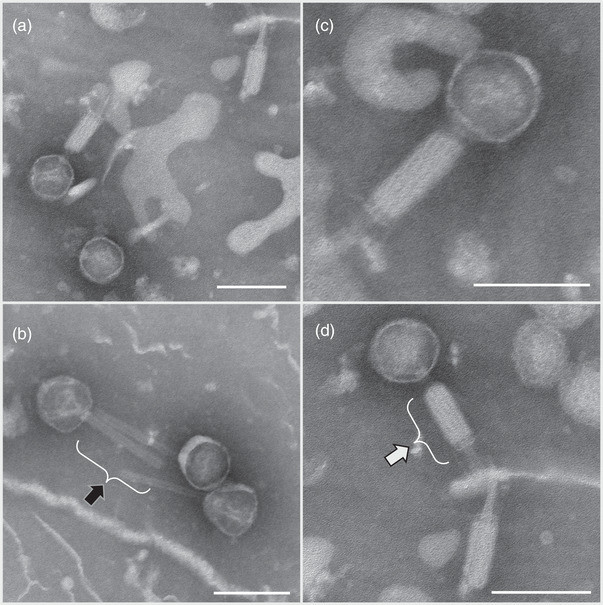
*Aggregatibacter* phage S1249 displays a contractile tail under transmission electron microscopy (TEM). Morphologies of phages at different assembly stages are presented. (a) Virus heads, (b) heads with inner rigid tubes of tails, (c and d) virions. This phage virion is composed of an oblate icosahedral head (∼60–70 nm in diameter) and a tail (∼100 nm in length) with tail fibers. The tail structure is composed of an inner rigid tube (∼15 nm in diameter, shown by the black arrow) and an outer contractile sheath (∼30 nm in diameter, shown by the write arrow). Scale bar: 100 nm.

**FIGURE 2:**
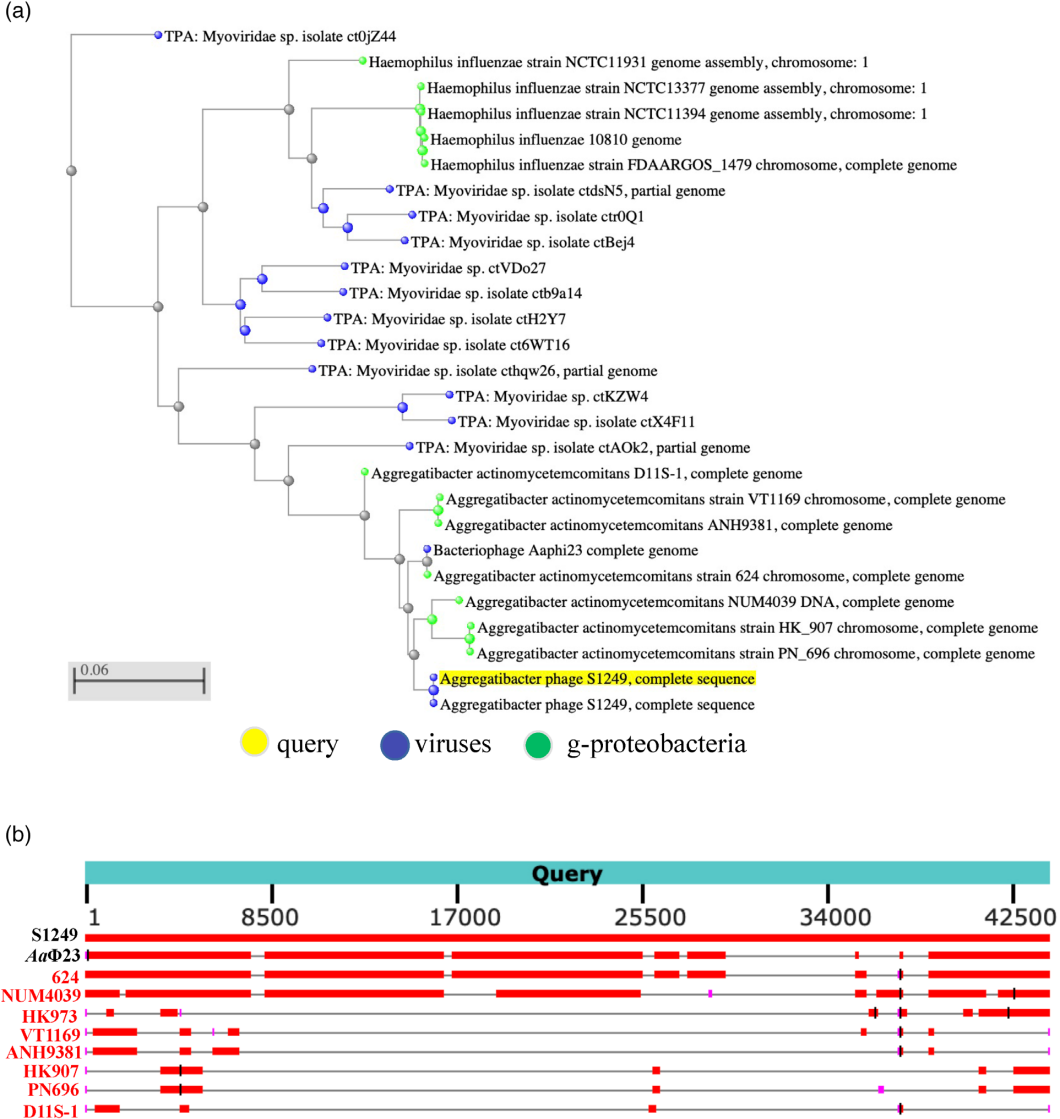
BLAST pairwise alignment using the phage S1249 sequence (GQ866233) against the database https://blast.ncbi.nlm.nih.gov/Blast.cgi. (a) Blast tree generated using neighbor‐joining method. Phage S1249 matches the DNA sequences of *Myoviridae* spp. (b) Distribution of the top 90 blast hits on 10 subjects of phages/prophage sequences identified from *A. actinomycetemcomitans*. Phage *Aa*Φ23 demonstrates 75% query coverage, and two *A. actinomycetemcomitans* strains, 624 and NUM4039, show 76% and 65% coverage, respectively. The difference is mainly located in the region encoding the regulation of DNA replication, modification, and recombination.

Initial sequencing of strain D11S‐1 (C. Chen et al., 2009) identified the presence of a 44 kbp phage DNA, which was noted as phage S1249. The examination of negatively stained isolated phages by TEM displayed a spheroid head with a diameter of approximately 60–70 nm and tail structures of ∼100 × 25 nm. The tail is composed of an inner rigid tube of ∼100 × 11 nm and an outer contractile sheath, as well as tail fibers (Figure 1). Morphologies of different stages of phage assemblage were identified, including oblate icosahedral heads, heads with inner tubes of the tail structures, and heads with complete tail structures (Figure 1). These features and the phage sequence alignment analysis against the database https://blast.ncbi.nlm.nih.gov/Blast.cgi (Figure 2) indicate that this tailed bacteriophage belongs to the family *Myoviridae*.

### 3.2 Bacterial DNA replication in the presence or absence of sera

**FIGURE 3:**
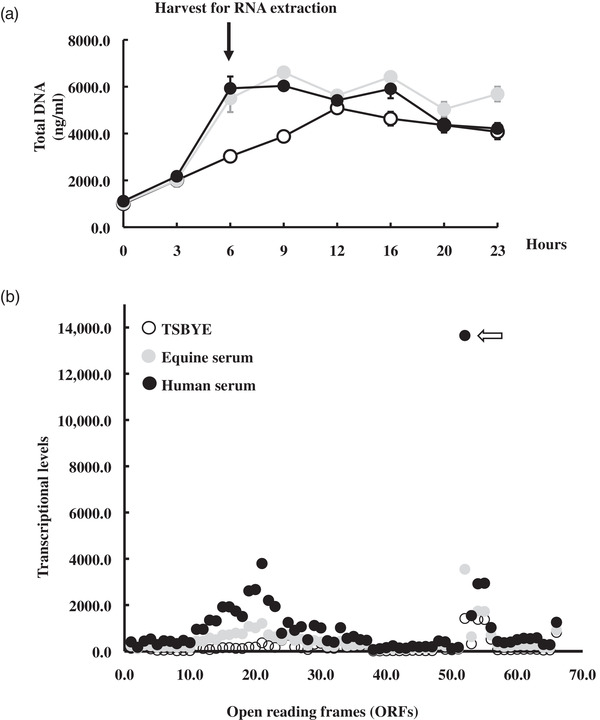
DNA replication of strain D11S‐1 and RNA‐seq analysis of phage S1249 under three growth conditions. (a) Total DNA replication and cell harvest for RNA extraction. Total DNA amounts were doubled in the presence of sera between 6 and 9 h. Cells were harvested at the 6‐h time point for RNA isolation. (b) RNA‐seq analysis. A total of 66 ORFs representing the whole phage genome are labeled with numbers on the X‐axis. Each dot represents an average transcription level of one gene based on duplicate independent experiments. Extensive transcriptional activation was observed, including 20% of the genes being upregulated greater than 10‐fold and 45% being upregulated between 5‐ and 10‐fold in the presence of human serum but not equine serum. One protein encoded by D11S_2259 was actively transcribed, especially in the presence of human serum (shown by hollow arrow).

Strain D11S‐1 is one of several *A. actinomycetemcomitans* strains that showed a distinct growth phenotype in the presence of human serum (Tang‐Siegel et al., 2016), featuring a second, rapid increase in turbidity after 9 h of incubation that reached a final optical density two to seven fold higher than other strains. This second increase in turbidity was notably associated with cell lysis (Tang‐Siegel et al., 2016). To determine if this phenomenon was caused by the induction of phage S1249, strain D11S‐1 was grown in either TSBYE or heat inactivated human or horse serum, and DNA was extracted every 3 h over a 24‐h period. No difference in the amount of DNA was observed among different growth media within the first 3 h of growth (Figure 3a). However, after 3 h of incubation in medium containing either human or horse serum, an approximately doubled amount of DNA was observed between the 6‐ and 9‐h time points, which subsequently declined to a level similar to that observed in TSBYE alone by 12 h and beyond (Figure 3a). Based on this experiment, RNA was extracted at the 6‐h time point to determine if phage DNA is represented at this kinetic time point.

### 3.3 Transcriptional analysis of phage S1249 genes in the presence of sera

Bacterial cells were harvested after 6 h of exposure to growth medium with or without sera for transcriptomic analysis. The entire phage genome, which is represented as a total of 66 ORFs (D11S_2208 to D11S_2273) (GenBank: GQ866233), was found to have transcriptional activities. Phage transcripts were found at higher levels in cells grown in the presence of either equine or human serum compared to cells grown in TSBYE alone (Figure 3b). However, the greatest increase in transcripts was observed in cells grown in the presence of human serum: 20% of genes were upregulated more than 10‐fold, whereas 45% were upregulated 5‐ to 10‐fold compared to TSBYE alone (Figure 3b). The transcripts for proteins from D11S_2215 to D11S_2230 encoding structural proteins were most highly regulated, including an 18‐fold increase of the tail‐length tape measure protein (*tmp*, D11S_2220) and a 13‐fold increase of the baseplate protein (*bpp*, D11S_2215) in human serum compared to without serum. A protein with the most active transcription encoded by gene D11S_2259 was upregulated 10‐fold when the cells were grown in human serum (Figure 3b), which encodes a 52‐amino‐acid protein with unknown function(s).

### 3.4 Kinetics of phage release

**FIGURE 4:**
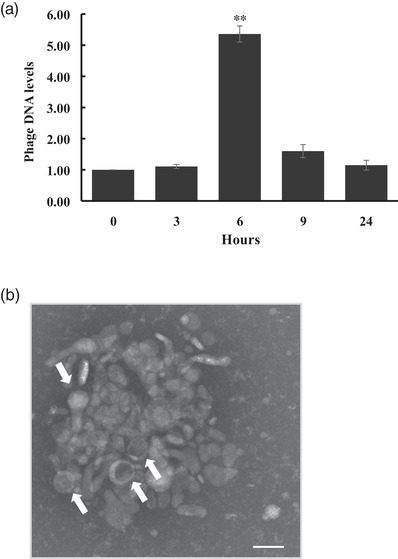
Phage S1249 virions released in the culture medium containing human serum form aggregates. (a) Phage DNA was quantified by PCR from the filtered, bacterial culture spent medium of D11S‐1. Bacterial spent media were collected after 0‐, 3‐, 6‐, 9‐ and 24‐h bacterial growth, and a fivefold increase in phage DNA was detected after 6‐h growth in the presence of human serum (**p < 0.01). (b) Phage virions (indicated by arrows) released from the bacterium formed aggregates in human serum. Scale bar: 100 nm.

To determine the kinetics of phage release, bacterial culture spent media were collected after 0‐, 3‐, 6‐, 9‐, and 24‐h exposure to human serum. Phage DNA was quantified by PCR of filtered spent media using two sets of primers corresponding to the *bpp* and *tmp* genes. Little to no difference in the amount of phage gene product was observed in the first 3 h of growth. However, a fivefold increase in the amount of targeted phage DNA was observed after 6 h of incubation, which subsequently declined at the 9‐ and 24‐h time periods (Figure 4a). Isolation and visualization of the released phage particles using TEM demonstrated that released phage virions formed aggregates in human serum (Figure 4b). This type of aggregation was not observed when phages were induced using mitomycin C (Figure 1).

### 3.5 Intracellular localization of phage S1249 DNA

**FIGURE 5:**
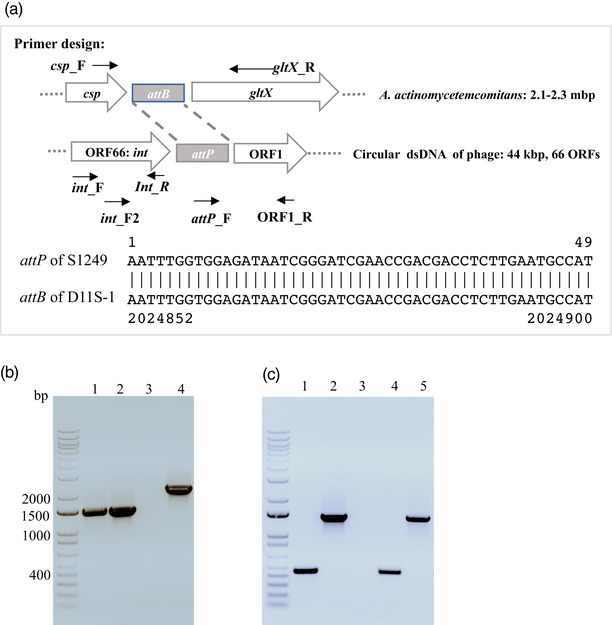
Intracellular location of phage DNA. (a) *attB* and *attP* sites: The *attP* site was identified from the phage located between ORF1 and ORF66 (the *int* gene), and the *attB* site was identified from *A. actinomycetemcomitans* between the *csp* and *gltX* genes. (b) Integration in strain D11S‐1. Lanes 1 and 2 were amplified using primers *csp_F* and *gltX_R* (1.5 kbp), and lanes 3 and 4 were amplified using primers *int_F* and *gltX_R (*2.2 kbp). The DNA of the phage‐free strain ATCC29523 was used as a control and amplified only with primers specific for the bacterium shown in lane 1 but not with primers *int_F* and *gltX_R* in lane 3. The DNA of D11S‐1 was displayed both without and with integrated phage DNA, as demonstrated by the 1.5‐ and 2.2‐kbp amplicons in lanes 2 and 4. (c) Circular dsDNA of phage S1249. To determine if phage DNA is circular when not integrated, PCR was performed using DNA isolated from bacteria, the isolated phage, and primers targeting the *attP* site, the first ORF1 and the last ORF66. Lanes 1 and 2: strain D11S‐1; lane 3: strain ATCC29523; lanes 4 and 5: virion S1249. PCR using primers *attP_*F and ORF1_R yielded a 450 bp product in both D11S‐1 (lane 1) and S1249 (lane 4), and PCR using *int*_F2 and ORF1_R yielded a 1.4 kbp amplicon in both D11S‐1 (lane 4) and S1249 (lane 5), indicating that a circular phage dsDNA is present in both infected strain D11S‐1 and the mature virus.

Lysogenic phages typically integrate into the bacterial chromosome aided by the *attP* site (Resch et al., 2004), and the *attP* site mediates the integration of phage DNA into the bacterial *attB* site of the chromosome, forming a prophage (Resch et al., 2004). As determined by sequencing, *Aggregatibacter* phage S1249 contains a 49‐bp *attP* site, and an identical 49‐bp *attB* site is found in D11S‐1 (C. Chen et al., 2009), which is located between the *csp* and *gltX* genes (Figure 5a). To determine if the phage DNA is integrated into the bacterial chromosome, primers corresponding to the *int* gene (D11S_2273, encoding a 348‐amino‐acid integrase for phage DNA recombination) located in the last ORF and primers (*csp_F* and *gltX_R*) corresponding to the genes surrounding the *attB* site located in the bacterial chromosome were used for PCR. A 2.2‐kbp fragment was generated using D11S‐1 DNA as the template, which corresponds to the size of the amplicon if the phage DNA was integrated into the chromosome (Figure 5b, lane 4). This amplicon was absent in a strain that does not contain phage (Figure 5b, lane 3). However, using a primer set of *csp_F* and *gltX_R*, a 1.5‐kbp amplicon (Figure 5b, lane 2) was also generated using D11S‐1 genomic DNA, and the same size amplicon was generated from an uninfected strain (Figure 5b, lane 1). The data suggest that both free and integrated phage DNA are present in the infected strain D11S‐1.

Our early study indicated that the circular form of phage S1249 DNA was detected in the infected strain D11S‐1 (C. Chen et al., 2009). To determine if the DNA is circular in virions, we isolated DNA from released phage virions. PCR was performed using DNA templates from either the infected bacterium or the virion. The primers *attP_*F and ORF1_R (D11S_2208) yielded a 450‐bp amplicon in the presence of phage dsDNA, while the primers *int*_F (D11S_2273, *int*) and ORF1_R yielded a 1.4‐kbp amplicon if the phage dsDNA was circular (Figures 5a,c). A 450‐bp amplicon was amplified from both strain D11S‐1 (Figure 5c, lane 1) and phage S1249 (Figure 5c, lane 4). A 1.4‐kbp amplicon was also amplified from both the infected bacterium (Figure 5c, lane 2) and the phage virion (Figure 5c, lane 5). The data suggest that a circular phage dsDNA is present in either the mature virus itself or its infected bacterial host.

### 3.6 Pseudolysogenic/lysogenic states of phage S1249 coexist in a newly generated infection model strain

**FIGURE 6:**
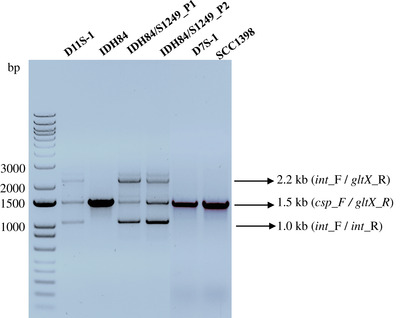
Genotype of phage S1249 in a generated infection model strain IDH84/S1249. Multiplex colony PCR was performed using four primers (*csp*_F, *gltX*_R, *int*_F, and *int_*R) targeting both bacterial and phage DNA at the same time. The 2.2 kb, 1.5 kb, and 1 kb bands represent phage DNA integrated, nonintegrated and infected by the phage. Cells from the same colony displayed both with and without phage DNA integrated into the bacterial chromosome in D11S‐1. The same genotype was observed in a newly generated, infected strain IDH84/S1249. IDH84: the parent strain without phage infection; IDH84/S1249_P1: single colony from the first passage after infection; IDH84/S1249_P2: single colony from the passaged strain of IDH84/S1249_P1. D7S‐1 and SCC1398 are two phage‐free strains demonstrating single bands only amplified by primers *csp*_F and *gltX*_R.

To further determine phage DNA location in bacterial cells, multiplex colony PCR was performed by using a single bacterial colony and four primers specific for bacterial and phage DNA (*csp*_F, *gltX*_R, *int*_F and *int_*R; described in Figure 5a and Table 2) at the same time. Three predominant amplicons were amplified from the colony of strain D11S‐1 (Figure 6): the 2.2‐kbp amplicon was amplified by primers *int*_F and *gltX*_R, indicating phage DNA integrated into the chromosome; the 1.5‐kbp fragment was amplified by primers *csp*_F and *gltX*_R, demonstrating phage DNA was not integrated into the chromosome; and the 1.0 kbp amplicon represents the contiguous form of the phage genome generated by primers *int*_F and *int_*R, indicating phage infection. Phage S1249 displayed both a truly lysogenic (with DNA integrated into the bacterial chromosome) and pseudolysogenic state (without DNA integrated into the bacterial chromosome) in strain D11S‐1 (Figure 6). The same genotype was also observed in a newly infected strain IDH84/S1249_P1 and the single in vitro passaged strain IDH84/S1249_P2 (Figure 6). The 1.5 kbp amplicons amplified using individual colonies of strains IDH84, DS7‐1, and SCC1389 were representative of uninfected cells that were only amplified by primers (*csp*_F and *gltX*_R) specific for the bacterium (Figure 6).

### 3.7 Phage specificity and serum sensitivity

**FIGURE 7:**
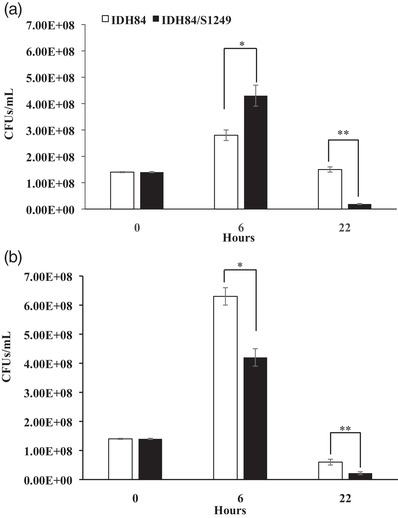
The infection model strain demonstrated human serum sensitivity. IDH84: the parent strain without phage infection; IDH84/S1249: strain IDH84 infected by phage S1249. (a) Grown in TSBYE. IDH84/S1249 demonstrated 1.5‐fold increased colony‐forming units (CFUs) compared to IDH84 after 6 h of growth in TSBYE. (b) Grown in 75% human serum. IDH84/S1249 demonstrated 33% and 63% reductions, respectively, in recovered CFUs compared to IDH84 after 6‐ and 22‐h exposure to 75% human serum. (*p < 0.05; **p < 0.01).

The isolated phage was used to infect different strains of *A. actinomycetemcomitans* to determine phage specificity. Two out of seven strains representing seven serotypes (a to f) demonstrated phage infectability, including strain IDH84 of serotype c and strain ATCC29523 of serotype a. The infected ATCC2953 strain only demonstrated lytic properties with 90% growth inhibition in the first 6‐h growth in TSBYE broth, and the infected cells lost the ability to divide and form colonies (Tang‐Siegel, 2022), whereas the generated strain IDH84/S1249 demonstrated a lysogenized phenotype, including better growth in TSBYE alone compared to IDH84 in the first 6 h (Figure 7a).

To determine the correlation between the presence of phage and increased sensitivity to human serum, the newly infected strain IDH84/S1249 and the parent strain IDH84 (phage‐free) were grown in TSBYE with/without 75% nonheated, type AB human serum. When grown in TSBYE alone, the infected strain demonstrated a 1.5‐fold increase in CFUs compared to the phage‐free strain after 6 h of growth (*p < 0.05), followed by an 87% reduction after 22 h (**p < 0.01, Figure 7a). In the presence of human serum, a 33% reduction after 6 h of exposure (*p < 0.05) and a 63% reduction in the recovered CFUs after 22 h (**p < 0.01) were observed in the infected strain compared to its isogenic phage‐free strain (Figure 7b).

## 4 DISCUSSION

Bacteriophages were first identified in *A. actinomycetemcomitans* four decades ago (Stevens et al., 1982). However, limited studies have been conducted since the first identification (Resch et al., 2004; Stevens et al., 2013; Szafrański et al., 2019; Willi et al., 1997). Phage S1249 displayed a spheroid head with an inner rigid tube and an outer contractile sheath, as well as tail fibers (Figure 1). The contractile tail features demonstrated by TEM suggest that S1249 is a member of the *Myoviridae* family, which was further confirmed by sequence alignment (Figure 2). Phage S1249 shares 75% similarity with phage *Aa*ϕ23 (Resch et al., 2004) and two prophages identified from *A. actinomycetemcomitans* strains: 624 and NUM4039 (Figure 2). The difference is mainly located in the region of 25,441‐38,392 (GQ866233: D11S_2244 to D11S_2263), encoding proteins regulating phage DNA replication and recombination, including anti‐termination protein Q D11S_2245 (upregulated 5.5‐fold in human serum), DNA adenine methylase D11S_2251 (upregulated 5.5‐fold), and two phage replication proteins D11S_2252 and _2253 (upregulated 6‐ and 8‐fold). The most transcriptionally active 52‐amino‐acid, uncharacterized protein (D11S_2259) is also located in this region (Figure 3b) and was not found in the previously identified *Aggregatibacter* phage *Aa*ϕ23.

The RNA‐seq data demonstrated active transcription of all 66 phage ORFs (Figure 3b), which contrasts with nondetected transcripts (data not shown) from plasmid S57 (24 kbp, Accession: GQ866235) and S25 (31 kbp, GQ866234) also found in the same strain, indicating that phage S1249 specifically responds to serum. Among the identified ORFs of this phage, those upregulated over 10‐fold were mainly phage morphology‐related proteins, which are normally upregulated in the late phase (Marrs & Howe, 1990; Nale et al., 2021). We also observed three to fivefold upregulation of other late‐phase genes, including the spanin protein Rz1 (D11S_2238) and Rz (D11S_2239) and endolysin (D11S_2240) (Figure 3b). These proteins are critical for phages to lyse bacterial double‐layer membranes and periplasmic peptidoglycan before escape from the bacterial cytosol once the progeny virions are fully assembled (Cahill & Young, 2019; Young, 1992). These observations support the hypothesis that some components in serum induce phase transition of the phage.

The PCR analysis of the bacterial culture spent medium indicated that the release of phage from the bacterium occurred approximately 6 h after incubation in human serum (Figure 4a). This is consistent with the total DNA synthesis of strain D11S‐1, which peaked at approximately 6 h (Figure 3a) after exposure to serum. This infected strain produced double the amount of DNA in human serum at this time point versus without serum, which is likely due to phage DNA replication. We also observed that phage virions released after serum induction formed aggregates (Figure 4b), which may contribute to the phenomenon of a second elevated optical density that we observed when this strain was incubated in human serum for more than 9 h (Tang‐Siegel et al., 2016).

Prophage S1249 was originally identified from strain D11S‐1, with suggested chromosome integration based on the *attB* site (C. Chen et al., 2009). In this study, we demonstrated the truly lysogenized phage DNA in strain D11S‐1 based on PCR analyses (Figures 5 and 6). However, we also found bacterial chromosomal DNA from the same colony without phage DNA integration, and circular phage DNA exists independently in the bacterial cells, suggesting that lysogenic/pseudolysogenic states coexist. Since the detection of circular phage DNA in strain D11S‐1 by PCR could be due to the presence of transient concatemers during rolling cycle phage replication (C. Chen et al., 2009; Weigel & Seitz, 2006), we further confirmed the circular dsDNA structure of this phage by PCR using purified virion DNA (Figure 5c). The DNA structure analysis of *Aa*ϕ23 using restriction enzymes and electron microscopy, however, demonstrated a linear DNA molecule with ∼1.6 kb terminal redundancy, indicating potential circular permutation (Willi & Meyer, 1998). Unlike S1249, *Aa*ϕ23 was found integrated into the chromosome of an *A. actinomycetemcomitans* strain (Willi & Meyer, 1998).

Consistent with D11S‐1, a newly infected strain IDH84/S1249 demonstrated the same genotype (Figure 6). The evidence of nonintegrated phage DNA and the absence of cell lysis suggests that this form of the phage exists in a pseudolysogenic state (Gabiatti et al., 2018; Łoś & Węgrzyn, 2012). This phage state is considered a stalled state caused by unfavorable growth conditions, including starvation, in which the phage does not synthesize virion particles but synchronizes the replication of the viral genome with the bacterial replication cycle (Łoś & Węgrzyn, 2012). An early study indicated that phages in the pseudolysogenic state were not inducible by using mutagenic agents, including mitomycin C, due to the lack of chromosome integration (Williamson et al., 2001). Our study, however, indicated here that the prophage S1249, which existed in both lysogenic/pseudolysogenic states, was inducible by using mitomycin C, as demonstrated (Figure 1). Nevertheless, our study suggests that this lysogenic/pseudolysogenic state may have a survival advantage for the phage, including switches to either lytic or lysogenic state based on the available growth conditions. A pseudolysogenic state has been reported to be required for certain phage‐mediated bacterial regulation (Cenens et al., 2013). Whether this state is related to phage induction in serum requires further investigation.

The rapid rise in antibiotic‐resistant strains is the impetus for the development of alternative antimicrobials. Antibiotic resistance mechanisms have widely spread, including the human oral microbiome (Caselli et al., 2020). Antibiotic‐resistant strains of *A. actinomycetemcomitans* have been identified (Akrivopoulou et al., 2017; Ardila & Bedoya‐García, 2020), and they acquire mechanisms including mobile rRNA methylase genes (Roe et al., 1996) for erythromycin resistance and the multidrug efflux pump system AcrAB‐TolC (MacA‐MacB‐TdeA) for multidrug resistance (Crosby & Kachlany, 2007; Narayanan et al., 2017). Bacteriophage therapy has resumed its position as an alternative antibacterial infection strategy to combat the rising threat of antibiotic resistance in the past decade (Hatfull et al., 2022; Suh et al., 2022; Uyttebroek et al., 2022) but is still limited to very few microorganisms. Therefore, it is important to identify and characterize phages/prophages and identify phages that can be potentially engineered for future therapeutic purposes. The S1249 phage investigated in this study may be a suitable candidate for this purpose.

In this study, we suggest that human serum induces the transition of the lysogenic phage S1249 into the lytic cycle that ultimately kills the bacterium. Additional investigation is needed to understand bacteriophage infections, virion replication, and release in this human oral pathobiont in in vivo environments. Knowledge obtained from these studies may result in a useful tool to effectively control bacterial infection in vivo.

## CONFLICT OF INTEREST

The authors declare no conflict of interest.

### PEER REVIEW

The peer review history for this article is available at https://publons.com/publon/10.1111/omi.12378.

## Data Availability

Important data are provided with the manuscript, and extra data are available upon request.

## References

[omi12378-bib-0001] Akrivopoulou, C. , Green, I. M. , Donos, N. , Nair, S. P. , & Ready, D. (2017). *Aggregatibacter actinomycetemcomitans* serotype prevalence and antibiotic resistance in a UK population with periodontitis. Journal of Global Antimicrobial Resistance, 10, 54–58. 28668698 10.1016/j.jgar.2017.03.011

[omi12378-bib-0002] Anton‐Vazquez, V. , Dworakowski, R. , Cannata, A. , Amin‐Youssef, G. , Gunning, M. , Papachristidis, A. , MacCarthy, P. , Baghai, M. , Deshpande, R. , Khan, H. , Byrne, J. , & Fife, A. (2022). 16S rDNA PCR for the aetiological diagnosis of culture‐negative infective endocarditis. Infection, 50(1), 243–249. 10.1007/s15010-021-01690-x 34490592

[omi12378-bib-0003] Ardila, C. M. , & Bedoya‐García, J. A. (2020). Antimicrobial resistance of *Aggregatibacter actinomycetemcomitans*, *Porphyromonas gingivalis* and *Tannerella forsythia* in periodontitis patients. Journal of Global Antimicrobial Resistance, 22, 215–218. 32169683 10.1016/j.jgar.2020.02.024

[omi12378-bib-0004] Asakawa, R. , Komatsuzawa, H. , Kawai, T. , Yamada, S. , Goncalves, R. B. , Izumi, S. , Fujiwara, T. , Nakano, Y. , Suzuki, N. , Uchida, Y. , Ouhara, K. , Shiba, H. , Taubman, M. A. , Kurihara, H. , & Sugai, M. (2003). Outer membrane protein 100, a versatile virulence factor of *Actinobacillus actinomycetemcomitans* . Molecular Microbiology, 50(4), 1125–1139. 10.1046/j.1365-2958.2003.03748.x 14622404

[omi12378-bib-0005] Cahill, J. , & Young, R. (2019). Phage lysis: Multiple genes for multiple barriers. Advances in Virus Research, 103, 33–70. 30635077 10.1016/bs.aivir.2018.09.003 PMC6733033

[omi12378-bib-0006] Casarin, R. C. , Ribeiro Edel, P. , Mariano, F. S. , Nociti, F. H., Jr. , Casati, M. Z. , & Gonçalves, R. B. (2010). Levels of *Aggregatibacter actinomycetemcomitans*, *Porphyromonas gingivalis*, inflammatory cytokines and species‐specific immunoglobulin G in generalized aggressive and chronic periodontitis. Journal of Periodontal Research, 45(5), 635–642. 10.1111/j.1600-0765.2010.01278.x 20546109

[omi12378-bib-0007] Caselli, E. , Fabbri, C. , D'Accolti, M. , Soffritti, I. , Bassi, C. , Mazzacane, S. , & Franchi, M. (2020). Defining the oral microbiome by whole‐genome sequencing and resistome analysis: The complexity of the healthy picture. BMC Microbiology, 20(1), 120. 10.1186/s12866-020-01801-y 32423437 PMC7236360

[omi12378-bib-0008] Cenens, W. , Mebrhatu, M. T. , Makumi, A. , Ceyssens, P. J. , Lavigne, R. , Van Houdt, R. , Taddei, F. , & Aertsen, A. (2013). Expression of a novel P22 ORFan gene reveals the phage carrier state in *Salmonella typhimurium* . PLoS Genetics, 9(2), e1003269. 10.1371/journal.pgen.1003269 23483857 PMC3573128

[omi12378-bib-0009] Chambers, S. T. , Murdoch, D. , Morris, A. , Holland, D. , Pappas, P. , Almela, M. , Fernandez‐Hidalgo, N. , Almirante, B. , Bouza, E. , Forno, D. , del Rio, A. , Hannan, M. M. , Harkness, J. , Kanafani, Z. A. , Lalani, T. , Lang, S. , Raymond, N. , Read, K. , Vinogradova, T. , … Chu, V. H. (2013). HACEK infective endocarditis: Characteristics and outcomes from a large, multi‐national cohort. PLoS One, 8(5), e63181. 10.1371/journal.pone.0063181 23690995 PMC3656887

[omi12378-bib-0010] Chen, C. , Kittichotirat, W. , Chen, W. , Downey, J. , Si, Y. , & Bumgarner, R. (2010). Genome sequence of naturally competent *Aggregatibacter actinomycetemcomitans* serotype a strain D7S‐1. Journal of Bacteriology, 192(10), 2643–2644. 10.1128/JB.00157-10 20348265 PMC2863555

[omi12378-bib-0011] Chen, C. , Kittichotirat, W. , Si, Y. , & Bumgarner, R. (2009). Genome sequence of *Aggregatibacter actinomycetemcomitans* serotype c strain D11S‐1. Journal of Bacteriology, 191(23), 7378–7379. 10.1128/JB.01203-09 19820097 PMC2786557

[omi12378-bib-0012] Chen, F. , Wang, K. , Stewart, J. , & Belas, R. (2006). Induction of multiple prophages from a marine bacterium: A genomic approach. Applied and Environmental Microbiology, 72(7), 4995–5001. 10.1128/AEM.00056-06 16820498 PMC1489376

[omi12378-bib-0013] Crosby, J. A. , & Kachlany, S. C. (2007). TdeA, a TolC‐like protein required for toxin and drug export in *Aggregatibacter (Actinobacillus) actinomycetemcomitans* . Gene, 388(1–2), 83–92. 10.1016/j.gene.2006.10.004 17116373 PMC1831674

[omi12378-bib-0014] Fine, D. H. , Patil, A. G. , & Loos, B. G. (2018). Classification and diagnosis of aggressive periodontitis. Journal of Periodontology, 89(Suppl 1), S103–S119. 10.1002/JPER.16-0712 29926947

[omi12378-bib-0015] Fine, D. H. , Schreiner, H. , & Velusamy, S. K. (2020). *Aggregatibacter*, a low abundance pathobiont that influences biogeography, microbial dysbiosis, and host defense capabilities in periodontitis: The history of a bug, and localization of disease. Pathogens, 9(3), 179. 10.3390/pathogens9030179 32131551 PMC7157720

[omi12378-bib-0016] Gabiatti, N. , Yu, P. , Mathieu, J. , Lu, G. W. , Wang, X. , Zhang, H. , Soares, H. M. , & Alvarez, P. J. J. (2018). Bacterial endospores as phage genome carriers and protective shells. Applied and Environmental Microbiology, 84(18), e01186–18. 10.1128/AEM.01186-18 30006404 PMC6121981

[omi12378-bib-0017] Hajishengallis, G. , & Chavakis, T. (2021). Local and systemic mechanisms linking periodontal disease and inflammatory comorbidities. Nature Reviews Immunology, 21(7), 426–440. 10.1038/s41577-020-00488-6 PMC7841384 33510490

[omi12378-bib-0018] Hatfull, G. F. , Dedrick, R. M. , & Schooley, R. T. (2022). Phage therapy for antibiotic‐resistant bacterial infections. Annual Review of Medicine, 73, 197–211. 10.1146/annurev-med-080219-122208 34428079

[omi12378-bib-0019] Konig, M. F. , Abusleme, L. , Reinholdt, J. , Palmer, R. J. , Teles, R. P. , Sampson, K. , Rosen, A. , Nigrovic, P. A. , Sokolove, J. , Giles, J. T. , Moutsopoulos, N. M. , & Andrade, F. (2016). *Aggregatibacter actinomycetemcomitans*‐induced hypercitrullination links periodontal infection to autoimmunity in rheumatoid arthritis. Science Translational Medicine, 8(369), 369ra176. 10.1126/scitranslmed.aaj1921 PMC5384717 27974664

[omi12378-bib-0020] Łoś, M. , & Węgrzyn, G. (2012). Pseudolysogeny. Advances in Virus Research, 82, 339–349. 22420857 10.1016/B978-0-12-394621-8.00019-4

[omi12378-bib-0021] Marrs, C. F. , & Howe, M. M. (1990). Kinetics and regulation of transcription of bacteriophage Mu. Virology, 174(1), 192–203. 10.1016/0042-6822(90)90068-3 2136777

[omi12378-bib-0022] Nale, J. Y. , Al‐Tayawi, T. S. , Heaphy, S. , & Clokie, M. R. J. (2021). Impact of phage CDHS‐1 on the transcription, physiology and pathogenicity of a *Clostridioides difficile* ribotype 027 strain, R20291. Viruses, 13(11), 2262. 10.3390/v13112262 34835068 PMC8619979

[omi12378-bib-0023] Narayanan, A. M. , Ramsey, M. M. , Stacy, A. , & Whiteley, M. (2017). Defining genetic fitness determinants and creating genomic resources for an oral pathogen. Applied and Environmental Microbiology, 83(14), e00797–17. 10.1128/AEM.00797-17 28476775 PMC5494627

[omi12378-bib-0024] Permpanich, P. , Kowolik, M. J. , & Galli, D. M. (2006). Resistance of fluorescent‐labelled *Actinobacillus actinomycetemcomitans* strains to phagocytosis and killing by human neutrophils. Cellular Microbiology, 8(1), 72–84. 10.1111/j.1462-5822.2005.00601.x 16367867

[omi12378-bib-0025] Resch, G. , Kulik, E. M. , Dietrich, F. S. , & Meyer, J. (2004). Complete genomic nucleotide sequence of the temperate bacteriophage Aa Phi 23 of *Actinobacillus actinomycetemcomitans* . Journal of Bacteriology, 186(16), 5523–5528. 10.1128/JB.186.16.5523-5528.2004 15292156 PMC490939

[omi12378-bib-0026] Roe, D. E. , Weinberg, A. , & Roberts, M. C. (1996). Mobile rRNA methylase genes coding for erythromycin resistance in *Actinobacillus actinomycetemcomitans* . Journal of Antimicrobial Chemotherapy, 37(3), 457–464. 10.1093/jac/37.3.457 9182102

[omi12378-bib-0027] Sørensen, M. C. , Gencay, Y. E. , Birk, T. , Baldvinsson, S. B. , Jäckel, C. , Hammerl, J. A. , Vegge, C. S. , Neve, H. , & Brøndsted, L. (2015). Primary isolation strain determines both phage type and receptors recognised by *Campylobacter jejuni* bacteriophages. PLoS One, 10(1), e0116287. 10.1371/journal.pone.0116287 25585385 PMC4293142

[omi12378-bib-0028] Stevens, R. H. , de Moura Martins Lobo Dos Santos, C. , Zuanazzi, D. , de Accioly Mattos, M. B. , Ferreira, D. F. , Kachlany, S. C. , & Tinoco, E. M. (2013). Prophage induction in lysogenic *Aggregatibacter actinomycetemcomitans* cells co‐cultured with human gingival fibroblasts and its effect on leukotoxin release. Microbial Pathogenesis, 54, 54–59. 23022667 10.1016/j.micpath.2012.09.005

[omi12378-bib-0029] Stevens, R. H. , Hammond, B. F. , & Lai, C. H. (1982). Characterization of an inducible bacteriophage from a leukotoxic strain of *Actinobacillus actinomycetemcomitans* . Infection and Immunity, 35(1), 343–349. 10.1128/iai.35.1.343-349.1982 7054125 PMC351035

[omi12378-bib-0030] Suh, G. A. , Lodise, T. P. , Tamma, P. D. , Knisely, J. M. , Alexander, J. , Aslam, S. , Barton, K. D. , Bizzell, E. , Totten, K. M. C. , Campbell, J. L. , Chan, B. K. , Cunningham, S. A. , Goodman, K. E. , Greenwood‐Quaintance, K. E. , Harris, A. D. , Hesse, S. , Maresso, A. , Nussenblatt, V. , Pride, D. , … Patel, R. (2022). Considerations for the use of phage therapy in clinical practice. Antimicrobial Agents and Chemotherapy, 66(3), e0207121. 10.1128/aac.02071-21 35041506 PMC8923208

[omi12378-bib-0031] Sundqvist, G. , & Johansson, E. (1982). Bactericidal effect of pooled human serum on *Bacteroides melaninogenicus*, *Bacteroides asaccharolyticus* and *Actinobacillus actinomycetemcomitans* . Scandinavian Journal of Dental Research, 90(1), 29–36. 6123149 10.1111/j.1600-0722.1982.tb01521.x

[omi12378-bib-0032] Szafrański, S. P. , Kilian, M. , Yang, I. , Bei der Wieden, G. , Winkel, A. , Hegermann, J. , & Stiesch, M. (2019). Diversity patterns of bacteriophages infecting *Aggregatibacter* and *Haemophilus* species across clades and niches. The ISME Journal, 13(10), 2500–2522. 10.1038/s41396-019-0450-8 31201356 PMC6776037

[omi12378-bib-0033] Tang, G. , Ruiz, T. , Barrantes‐Reynolds, R. , & Mintz, K. P. (2007). Molecular heterogeneity of EmaA, an oligomeric autotransporter adhesin of *Aggregatibacter (Actinobacillus) actinomycetemcomitans* . Microbiology, 153(8), 2447–2457. 10.1099/mic.0.2007/005892-0 17660409

[omi12378-bib-0035] Tang‐Siegel, G. G. (2022). Characterization of *Aggregatibacter* phage S1249: Lysogenic and lytic phenotype switches. Journal of Dental Research, 101, abstract 0298. https://iadr.abstractarchives.com/abstract/51am‐3652616/characterization‐of‐aggregatibacter‐phage‐s1249‐lysogenic‐and‐lytic‐phenotype‐switches

[omi12378-bib-0034] Tang‐Siegel, G. G. , Bumgarner, R. , Ruiz, T. , Kittichotirat, W. , Chen, W. , & Chen, C. (2016). Human serum‐specific activation of alternative sigma factors, the stress responders in *Aggregatibacter actinomycetemcomitans* . PLoS One, 11(8), e0160018. 10.1371/journal.pone.0160018 27490177 PMC4973924

[omi12378-bib-0036] Tang‐Siegel, G. G. , Chen, C. , & Mintz, K. P. (2021). Phage induction by human serum in *Aggregatibacter actinomycetemcomitans* . Journal of Dental Research, 100, abstract 2048. https://iadr.abstractarchives.com/abstract/21iags‐3567371/phage‐induction‐by‐human‐serum‐in‐aggregatibacter‐actinomycetemcomitans

[omi12378-bib-0037] Uyttebroek, S. , Chen, B. , Onsea, J. , Ruythooren, F. , Debaveye, Y. , Devolder, D. , Spriet, I. , Depypere, M. , Wagemans, J. , Lavigne, R. , Pirnay, J. P. , Merabishvili, M. , De Munter, P. , Peetermans, W. E. , Dupont, L. , Van Gerven, L. , & Metsemakers, W. J. (2022). Safety and efficacy of phage therapy in difficult‐to‐treat infections: A systematic review. The Lancet Infectious Diseases, 5(7), 1049. 10.1016/S1473-3099(21)00612-5 35248167

[omi12378-bib-0038] Weigel, C. , & Seitz, H. (2006). Bacteriophage replication modules. Fems Microbiology Review, 30(3), 321–381. 10.1111/j.1574-6976.2006.00015.x 16594962

[omi12378-bib-0039] Willi, K. , & Meyer, J. (1998). DNA analysis of temperate bacteriophage *Aa*(phi)23 isolated from *Actinobacillus actinomycetemcomitans* . Molecular & General Genetics, 258(4), 323–325. 10.1007/s004380050737 9648735

[omi12378-bib-0040] Willi, K. , Sandmeier, H. , Asikainen, S. , Saarela, M. , & Meyer, J. (1997). Occurrence of temperate bacteriophages in different *Actinobacillus actinomycetemcomitans* serotypes isolated from periodontally healthy individuals. Oral Microbiology and Immunology, 12(1), 40–46. 10.1111/j.1399-302X.1997.tb00365.x 9151643

[omi12378-bib-0041] Williamson, S. J. , McLaughlin, M. R. , & Paul, J. H. (2001). Interaction of the PhiHSIC virus with its host: Lysogeny or pseudolysogeny? Applied And Environmental Microbiology, 67(4), 1682–1688. 10.1128/AEM.67.4.1682-1688.2001 11282621 PMC92785

[omi12378-bib-0042] Yamaguchi, N. , Kawasaki, M. , Yamashita, Y. , Nakashima, K. , & Koga, T. (1995). Role of the capsular polysaccharide‐like serotype‐specific antigen in resistance of *Actinobacillus actinomycetemcomitans* to phagocytosis by human polymorphonuclear leukocytes. Infection and Immunity, 63(12), 4589–4594. 10.1128/iai.63.12.4589-4594.1995 7591110 PMC173659

[omi12378-bib-0043] Young, R. (1992). Bacteriophage lysis: Mechanism and regulation. Microbiological Reviews, 56(3), 430–481. 10.1128/mr.56.3.430-481.1992 1406491 PMC372879

